# LATS1/2 loss promote tumor immune evasion in endometrial cancer through downregulating MHC-I expression

**DOI:** 10.1186/s13046-024-02979-z

**Published:** 2024-02-21

**Authors:** Qianlan Yang, Zehen Lv, Mengfei Wang, Mengwen Kong, Cheng Zhong, Kun Gao, Xiaoping Wan

**Affiliations:** 1 Department of Gynecology, Shanghai First Maternity and Infant Hospital, School of Medicine, Tongji University, Shanghai, 200092 China; 2 Department of Clinical Laboratory, Shanghai First Maternity and Infant Hospital, School of Medicine, Tongji University, Shanghai, 200092 China; 3 Shanghai Key Laboratory of Maternal and Fetal Medicine, Shanghai First Maternity and Infant Hospital, Shanghai, 200092 China

**Keywords:** Endometrial cancer, Immune evasion, MHC-I, LATS1/2, STAT1

## Abstract

**Background:**

LATS1/2 are frequently mutated and down-regulated in endometrial cancer (EC), but the contributions of LATS1/2 in EC progression remains unclear. Impaired antigen presentation due to mutations or downregulation of the major histocompatibility complex class I (MHC-I) has been implicated in tumor immune evasion. Herein, we elucidate the oncogenic role that dysregulation of LATS1/2 in EC leads to immune evasion through the down-regulation of MHC-I.

**Methods:**

The mutation and expression as well as the clinical significance of LATS1/2 in EC was assessed in the TCGA cohort and our sample cohort. CRISPR-Cas9 was used to construct knockout cell lines of LATS1/2 in EC. Differentially expressed genes were analyzed by RNA-seq. The interaction between LATS1/2 and STAT1 was verified using co-immunoprecipitation and GST pull-down assays. Mass spectrometry, in vitro kinase assays, ChIP-qPCR, flow cytometry, immunohistochemistry, immunofluorescence and confocal microscopy were performed to investigate the regulation of LATS1/2 on MHC-I through interaction with and phosphorylate STAT1. The killing effect of activated PBMCs on EC cells were used to monitor anti-tumor activity.

**Results:**

Here, we demonstrate that LATS1/2 are frequently mutated and down-regulated in EC. Moreover, LATS1/2 loss was found to be associated with a significant down-regulation of MHC-I, independently of the Hippo-YAP pathway. Instead, LATS1/2 were found to directly interact with and phosphorylate STAT1 at Ser727, a crucial transcription factor for MHC-I upregulation in response to interferon-gamma (IFN-γ) signaling, to promote STAT1 accumulating and moving into the nucleus to enhance the transcriptional activation of IRF1/NLRC5 on MHC-I. Additionally, the loss of LATS1/2 was observed to confer increased resistance of EC cells to immune cell-mediated killing and this resistance could be reversed by over-expression of MHC-I.

**Conclusion:**

Our findings indicate that dysregulation of LATS1/2 in EC leads to immune evasion through the down-regulation of MHC-I, leading to the suppression of infiltrating activated CD8 + T cells and highlight the importance of LATS1/2 in IFN-γ signaling-mediated tumor immune response, suggesting that LATS1/2 is a promising target for immune checkpoint blockade therapy in EC.

**Supplementary Information:**

The online version contains supplementary material available at 10.1186/s13046-024-02979-z.

## Introduction

Endometrial cancer (EC) ranks as the fourth most common cancer and the sixth leading cause of cancer-related mortality in women worldwide, as well as the only gynecological malignancy with rising mortality [1, 2]. Molecular subtyping and targeted therapy have emerged as promising approaches for EC management that aim to personalize treatment and improve patient outcomes. Molecular subtyping classifies EC into distinct subgroups based on the underlying molecular alterations; this classification promotes a more refined understanding of the disease and can guide treatment decisions. The most widely recognized molecular subtypes of EC include POLE ultra-mutated, microsatellite instability hypermutated, copy number high, and copy number low subtypes [3]. However, several challenges, including tumor heterogeneity, resistance, optimal treatment sequencing, and accessibility of targeted therapies, need to be addressed to fully realize the potential of these approaches in improving patient outcomes [4].

Major histocompatibility complex class I (MHC-I) proteins in humans are termed human leucocyte antigen I (HLA-I) and divided into classical and non-classical HLA-I subtypes. Classical type I HLA molecules (HLA-A, HLA-B, and HLA-C) play a critical role in the immune response against tumor cells by presenting tumor-specific antigens on the surface of cells for recognition by cytotoxic T cells [5]. The recognition of MHC-I-peptide complexes by cytotoxic T cells is mediated by the T cell receptor (TCR), which interacts with both the peptide and the MHC-I molecule. This interaction triggers a cascade of signaling events within T cells, leading to the activation of cytotoxic effector functions and the elimination of tumor cells [6, 7]. Tumor cells employ various strategies to downregulate the expression of MHC-I molecules. One of the common mechanisms involves genetic alterations, such as mutations or deletions in the genes encoding MHC-I molecules [8]. Additionally, tumor cells can utilize epigenetic modifications to silence MHC-I gene expression [7, 9]. Through these genetic and epigenetic modifications, tumor cells effectively dampen MHC-I expression, making themselves less visible to cytotoxic T cells and enabling immune evasion. The transcriptional regulation of MHC-I involves three major transcription binding sites: an Enhancer A region, which can be recognized by NF-κB [10]; an interferon-stimulated response element (ISRE), which can be bound by interferon regulatory factor 1 (IRF1) [11, 12]; an SXY-module, which is recognized by the NOD-like receptor family CARD domain containing 5 (NLRC5) [13, 14]. Upon stimulation by IFN-γ, a cytokine produced by immune cells, the Janus kinase-signal transducer and activator of transcription (JAK-STAT) signaling pathway is activated. This leads to the phosphorylation and dimerization of STAT1 which translocates to the nucleus, binds to the promoters of IRF1 and NLRC5, and transactivates their expression [12, 15]. This binding enables the recruitment of co-activators and other transcription factors to drive the transcriptional activation of MHC-I [6]. The phosphorylation of STAT1 at Yyr701 and Ser727 has been documented to be crucial for STAT1 transcriptional activity [16, 17]. JAK1/2 is responsible for STAT1 Yyr701 phosphorylation during STAT1 forming dimers, but the phosphorylation mechanism of STAT1 Ser727 is still unclear [18, 19], also several protein kinases, including p38, protein kinase C (PKC)-δ and CDK8, were reported to mediate different stimulant-induced STAT1 Ser727 phosphorylation [20–22].

The AGC family protein kinases LATS1 and LATS2 are key components of the Hippo signaling pathway, which is involved in controlling organ size and preventing tumorigenesis [23]. LATS1/2 exert tumor-suppressive functions by phosphorylating and inactivating the Yes-associated protein (YAP) and the transcriptional co-activator with PDZ-binding motif (TAZ), which are transcriptional co-activators promoting cell proliferation and survival. While the primary role of LATS1/2 is well-established as key components of the Hippo signaling pathway, recent studies have revealed additional diverse molecular functions of LATS1/2 beyond their involvement in Hippo-YAP signaling [24]. LATS1/2 kinases have been implicated in various cellular processes, including cell cycle control, DNA damage response, mitosis, cellular polarity, and cell migration. Genetic alterations in LATS1/2, including mutations, deletions, and promoter hypermethylation, have been identified in several types of human cancer [24]. These alterations can result in the loss of LATS1/2 function and the subsequent dysregulation of downstream signaling.

In this study, we present evidence of the frequent occurrence of mutations and downregulation of LATS1/2 in EC. To elucidate the molecular pathways that contribute to LATS1/2 loss-driven EC tumorigenesis, we conducted RNA-sequencing (RNA-seq) analysis to compare the gene expression profiles of LATS1/2 knockout (KO) EC cells generated by CRISPR/Cas9 gene editing with those of the parental cells. Surprisingly, we discovered a significant downregulation of MHC-I subtypes (HLA-A, HLA-B, and HLA-C) upon LATS1/2 loss, independently of the well-known Hippo-YAP pathway. Mechanistically, we demonstrated that LATS1/2 interacts with STAT1 and mediates its phosphorylation at Ser727, which is vital for IFN-γ-induced upregulation of MHC-I in both human and murine cells. Our findings highlight a conserved mechanism utilized by EC to exploit the loss of LATS1/2 function, enabling immune evasion.

## Methods

### Database acquisition and analyzation

The somatic mutation data, and clinical information of ECs were obtained through cBioPortal (https://www.cbioportal.org/). The UALCAN analysis (http://ualcan.path.uab.edu/) was used to obtain expression profiles of normal endometrial tissues and EC. The quantitative proteomics data for EC was obtained from the CPTAC database (proteomics.cancer.gov/programs/cptac). The targeted sequencing panels (OncoScreen®, Burning Rock Biotech) were used to assess the mutation status of 520 genes in a cohort of 99 primary EC specimens.

### EC specimen collections

The experimental protocols were approved by the hospital’s Human Subjects Review Board, and informed consent was obtained from each patient. A total of 99 primary EC specimens were obtained from our hospital between January 2010 and January 2018 for targeted sequencing. Additionally, 42 paraffin-embedded EC specimens and 4 normal endometrial specimens were obtained from our hospital between January 2018 and December 2021. Each patient included in the study was diagnosed with EC and tissue biopsies were histologically confirmed. None of the patients had previously undergone endocrine therapy, radiotherapy, chemotherapy, or surgery.

### IHC analysis

IHC was conducted based on the methods outlined in our previous study. We prepared all tissue slices, which were 4 mm in thickness, from paraffin-embedded samples. Two pathologists, unaware of the clinical and pathological details, assessed these samples. The specimens were then rated using semi-quantitative immunoreactivity scales. The proportion of positive staining was categorized as: 0 for 0–5%, 1 for 6-25%, 2 for 26–50%, 3 for 51–75%, and 4 for over 75%. The intensity of the staining was graded as: 0 for none, 1 for weak, 2 for moderate, and 3 for strong. The final score for each sample was determined by multiplying the scores of both criteria. The antibodies used for the IHC analysis are listed in Table S3.

### Cell culture, transfection, and viral infection

293T, KLE, HEC-1 A, and CT26 cells were obtained from American Type Culture Collection and maintained in DMEM supplemented with 10% (v/v) FBS and cultured in a humidified incubator at 37 °C with 5% CO_2_. Transient transfections were performed using Lipofectamine 2000. For lentivirus transfection, shRNA knockdown plasmids or pLVX-TetOne-Puro plasmids and virus packing constructs were transfected into 293 T cells. The viral supernatant was collected 48 h after transfection. Cells were infected with the virus using polybrene and cultured in 1 µg/ml puromycin for three days. Tet-on expression of exogenous LATS1/2-WT and their mutants was induced by treatment with doxycycline (10 ng/ml) for 24 h. The shRNA sequences used are listed in Table S4.

### RT-qPCR assays

Cells were processed to obtain total RNA using Trizol (Thermo Fisher Scientific). The cDNA was then produced through reverse transcription, utilizing the PrimeScript RT Master kit (TAKARA) following the guidelines provided by the manufacturer. The PCR amplification step was carried out with the AceQ Universal SYBR qPCR Master Mix Kit (Vazyme). All quantifications were normalized to the level of the endogenous control GAPDH. The primer sequences used for RT-qPCR analysis are listed in Tables S5.

### CRISPR-Cas9 mediated gene knock out of stable cell generations

The pX459 plasmid was employed to clone guide oligos targeting *LATS1* or *LATS2* genes. Cells were plated and underwent overnight transfection with the pX459 constructs. Post 48 h, puromycin (1 µg/ml) was applied to screen cells for 3 days. Surviving cells were then seeded into a 96-well plate using a limited dilution method to derive a single-cell lineage. Gene KO clones were identified using western blotting and confirmed through Sanger sequencing. The specific sequences of the gene-targeting single guide RNAs (sgRNAs) are listed in Table S5.

### RNA-seq and data analysis

Total RNA was extracted from parental cells and LATS1/2-KO KLE cells by Trizol reagent and then subjected to PE150 HiSeq using the Illumina NovaSeq 6000 sequencing platform, which was performed by ApexBio Technology LLC (Shanghai China). Differentially expressed genes (DEGs) were determined using DEseq2 (for sample with replications) with a cut-off value of log2|fold-change|≥0.25, *p* < 0.05. DEGs were analyzed using KEGG (https://www.kegg.jp/) pathway enrichment analysis. GSEA was performed by the function in package ClusterProfiler with a gene list sorted by log2 fold-change. Statistical significance was determined at *P* < 0.05. The RNA-seq data have been deposited to the Gene Expression Omnibus (GEO) with the dataset identifier GSE241847.

### Immunofluorescence (IF) and confocal microscopy

Cells were seeded onto chamber slides and stabilized using 4% paraformaldehyde at room temperature for 20 min. Following this, they were permeabilized with 0.1% Triton X-100 in PBS for 15 min at room temperature, then washed with PBS, blocked with 3% BSA at room temperature for 1 h, and incubated with primary antibodies in PBS at 4 °C overnight. After washing with PBS, the cells were exposed to fluorescent-tagged secondary antibodies for 1 h at room temperature in the dark. The cell nuclei were stained with DAPI at room temperature for 10 min. In the end, the cells were visualized and imaged using a fluorescence microscope or a confocal laser scanning microscope (Zeiss). The antibodies used for IF analysis are listed in Table S3.

### Cytokines detection with ELISA method

PBMCs were isolated from fresh whole blood of healthy female volunteers using Ficoll density gradient centrifugation. The isolated PBMCs were then cultured in RPMI-1640 medium and stimulated with PHA (1 µg/ml) for 48 h, with the addition of human recombinant IL-2. After activation, the PBMCs were co-cultured with parental or LATS1/2-KO KLE cells in 6-well plates at a ratio of 2:1, for 24 h. Following co-culture, the PBMCs were removed from the supernatant, and the concentrations of TNF-α and Granzyme B in the supernatant were measured using ELISA kits according to the manufacturer’s instructions.

### Cell apoptosis analysis with flow cytometry

An Annexin V-APC/7-ADD Apoptosis Detection Kit (KeyGEN Bio TECH) was used to measure the rate of apoptosis according to the manufacturer’s protocol. The activated PBMCs were co-cultured with parental or LATS1/2-KO KLE cells in 6-well plates at a ratio of 2:1, for 24 h. After the PBMCs were removed from the supernatant, the KLE cells were washed twice with cold PBS. The KLE cells were then digested with 0.25% trypsin (without EDTA), resuspended in binding buffer at a concentration of 1 × 10^6 cells/ml, and incubated with APC Annexin V and 7-ADD at room temperature for 15 min after mixing. Finally, the apoptotic rate was detected using a flow cytometer and analyzed using FlowJo software (version 10.8). To detect the NK cells-mediated killing on tumor cells, the NK cells were isolated from PBMCs using MojoSort™ Human NK Cell Isolation Kit according to the manufacturer’s instructions and co-cultured with parental or LATS1/2-KO KLE cells in 6-well plates at a ratio of 2:1, for 24 h. Then, the KLE cells were collected and incubated with APC Annexin V and 7-ADD to detect the apoptotic rate.

### Cell proliferation assays

Cell proliferation was determined using a CCK-8 Kit (Dojindo) following the manufacturer’s instructions. The activated PBMCs were co-cultured with parental or LATS1/2-KO KLE cells in 96-well plates at a ratio of 1: 1 for 48 h. The PBMCs were then removed from the supernatant and the KLE cells were washed twice with cold PBS. Then, 10 µl CCK-8 solution was added to the cell culture and the cells were incubated for 2 h. The resulting color was measured at 450 nm using a Multimode Plate Reader (Molecular Devices). Each treatment was performed in triplicate, and the experiments were repeated over three times.

### ChIP-qPCR assays

Chromatin Immunoprecipitation (ChIP) was carried out on both parental and LATS1/2-KO KLE cells. These cells were stabilized using 1% formaldehyde at room temperature for 10 min, followed by halting the cross-linking process with 1.25 M glycine for 5 min. After a double rinse with ice-cold PBS, the cells were collected into tubes through scraping and then sonicated until the cell lysate became transparent and the chromatin broke down to the target size range of 100–500 bp. From each sample, 20 µL of diluted DNA was set aside to measure the input DNA. The remaining solutions underwent immunoprecipitation by incubating overnight at 4 °C with either an anti-STAT1 antibody or IgG as a control. The protein-DNA complexes were subsequently isolated using protein A/G-Magnetic beads over 2 h. The samples underwent sequential washes in low-salt wash buffer (0.1% SDS, 1% Triton X-100, 2mM EDTA, 20mM Tris-HCl (pH 8.1), 150mM NaCl), high-salt wash buffer (0.1% SDS, 1% Triton X-100, 2mM EDTA, 20mM Tris-HCl (pH 8.1), 500mM NaCl), LiCl wash buffer (0.25 M LiCl, 1% IGEPAL-CA630, 1% sodium deoxycholate, 1mM EDTA, 10mM Tris-HCl (pH 8.1)), and twice in TE buffer (10mM Tris-HCl, 1mM EDTA, pH 8.0). Finally, the protein-DNA complexes were prepared for qPCR evaluation. The qPCR primer sequences are listed in Table S5.

### Co-IP assays

The interactions between LATS1 or LATS2 and STAT1 were investigated using Co-IP assays. Flag-LATS1 or LATS2 and myc-STAT1 were co-expressed in 293T cells for 24 h, and then the cells were lysed in immunoprecipitation buffer (50mM Tris-HCl pH 8.0, 150mM NaCl, 0.05mM EDTA, 1% NP40 and 10% glycerol). The cell lysates were immunoprecipitated overnight with anti-FLAG-beads (Smart-Lifesciences). For endogenous Co-IP assays, the KLE cells were lysed in immunoprecipitation buffer and the lysate was pre-cleared with protein-A/G agarose for 2 h at 4 °C. The supernatant was immunoprecipitated overnight with anti-LATS1, LATS2, or STAT1 antibody and subsequently immunoprecipitated with protein-A/G agarose for 2 h at 4 °C. The pellets were washed four times with immunoprecipitation buffer, resuspended in sample buffer, and analyzed by SDS/PAGE (10% gel).

### GST pull-down assays

pEGX-4T2 plasmids containing different constructs (GST, GST-STAT1 full length, or specific fragments) and pET28b plasmids encoding His-tagged LATS1 or LATS2 were separately introduced into *Escherichia coli* BL21. The transformed cells were then cultured at 37 °C for 5 h. Protein expression was induced by adding 0.1mM IPTG, followed by further culturing at 16 °C for 16 h. After culturing, the cells were harvested, sonicated in cold PBS, and purified using either Glutathione Agarose beads (Thermo) or Ni-NTA Agarose beads (Qiagen) according to the manufacturer’s instructions. For GST pull-down assays, the Glutathione Agarose beads were used to purify GST or GST-STAT1 recombinant proteins. Then the GST-beads or GST-STAT1-beads was incubated in lysates containing His-LATS1 or His-LATS2 proteins at 4 °C for 2 h. The final precipitates were dissolved in 1X SDS buffer and analyzed by WB and Coomassie blue staining to detect the presence of the desired proteins.

### In vitro kinase assays and mass spectrometry analysis

Induction method of the target proteins is described above. For purification of GST and GST-STAT1 recombinant proteins, Glutathione Agarose beads were used to combine GST or GST-STAT1 recombinant proteins at 4 °C for 2 h and the complex was eluted using glutathione (10mmol/L). For purification of His-LATS1 and His-LATS2 proteins, Ni-NTA Agarose beads were employed to combine His-LATS1 or His-LATS2 recombinant proteins at 4 °C for 2 h and the complex was eluted using imidazole (500mM). The purified recombinant kinase active His-LATS2 (480–1088 aa) or His-LATS1 (589–1130 aa) was mixed with purified recombinant GST-STAT1 fragments in the kinase buffer (20 Mm Tris-HCl [pH 7.5], 5mM MgCl_2_, 5mM MnCl_2_, 2mM DTT, 10µM ATP) and incubated at 30 °C for 30 min. The reaction was terminated by adding 2× SDS loading buffer and boiling at 95 °C for 5 min. SDS-PAGE was used to separate proteins, with gel bands cut out and subjected to mass-spectrometric sequencing. The proteomics data have been deposited to the ProteomeXchange Consortium via the PRIDE partner repository with the dataset identifier PXD043500.

### Mouse tumor implantation assays

Animal experiments were approved by the Ethics Review Committee for Animal Experimentation of Tongji University. Animals were housed in a pathogen-free environment with unrestricted access to food and water. Female BALB/c or BALB/c^nu/nu^ mice were obtained from Shanghai JieSiJie Laboratory Animals Co., LTD. For the experiments, 3 × 105 parental and LATS1/2-KO CT26 cells were subcutaneously injected into 6-week-old female BALB/c or BALB/c^nu/nu^ mice. Five mice were used for each data point, with one tumor per mouse. Tumor growth was monitored every 3 days in two dimensions using a digital caliper. Tumor volumes were calculated using the ellipsoid volume formula V = (L× W^2^)/2, where L is the length and W is the width. 21 days after tumor cell injection, the mice were euthanized and in vivo solid tumors were excised and weighed. Tumor tissues were divided, and a portion was subjected to formalin fixation and embedded in paraffin for IF analysis.

### Statistical analysis

Statistical analyses were conducted with the GraphPad Prism software (GraphPad Software). Data are presented as mean values ± SD, based on a minimum of three experimental repeats. For comparing two sets of data, the Student’s t-test was utilized, while for multiple sets, either One-way or Two-way ANOVA was employed, unless otherwise specified. Regarding statistical significance, * represents *p* < 0.05; ** represents *p* < 0.01; *** represents *p* < 0.001; **** represents *p* < 0.0001.

## Results

### LATS1/2 are frequently mutated and downregulated in EC

**Fig. 1:**
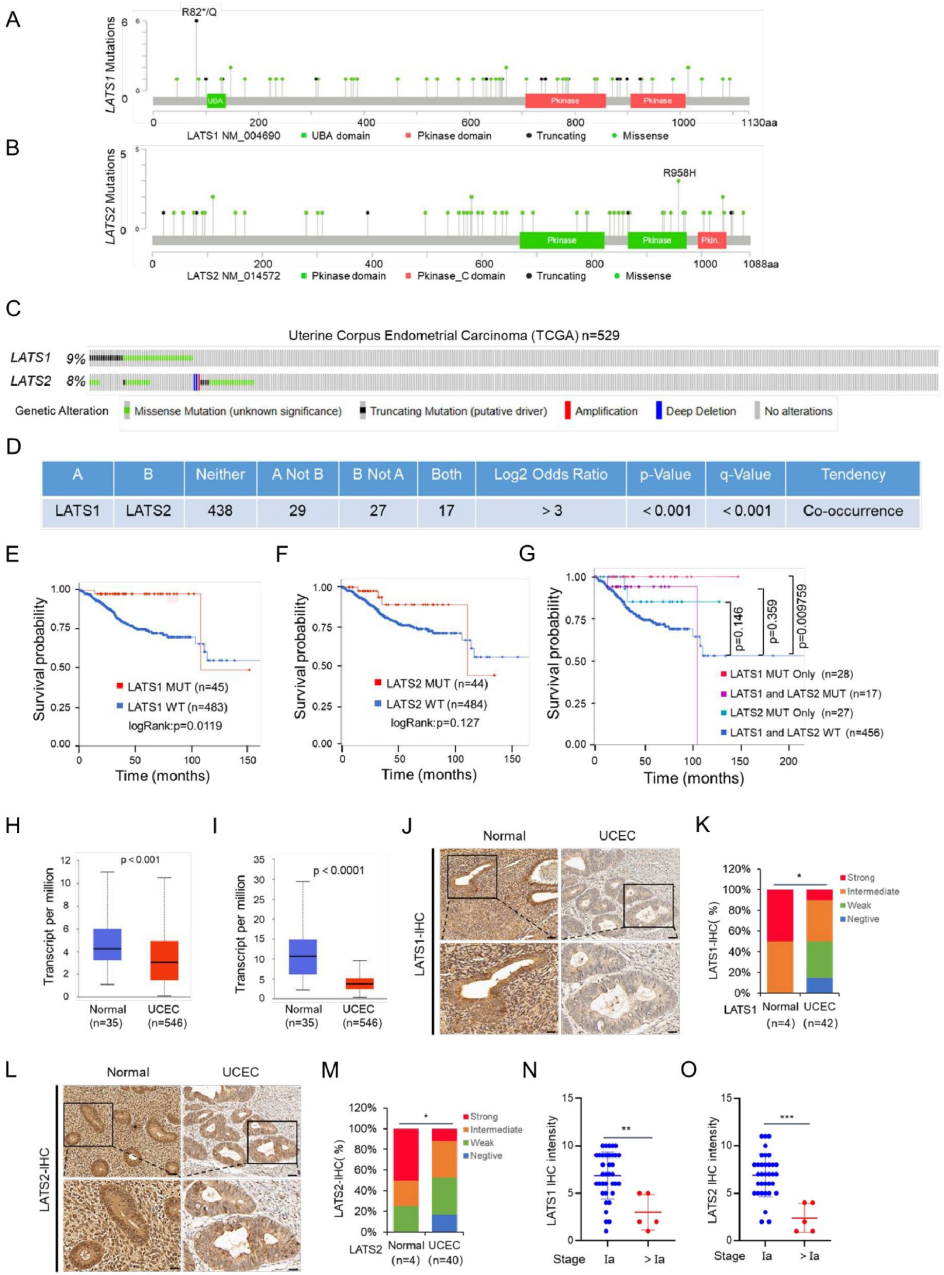
LATS1 and LATS 2 are frequently mutated and downregulated in patients with EC. **(A, B)** Schematics of LATS1 **(A)** and LATS2 **(B)** proteins showing the positions of individual somatic mutations identified in the TCGA EC cohort. **(C)** Co-occurrence of LATS1 and LATS2 mutations in the TCGA EC cohort. **(D)** Genetic alteration of LATS1 and LATS2 in the TCGA EC cohort. **(E, F)** Kaplan–Meier survival curves for patients with LATS1 **(E)** or LATS2 **(F)** mutated and wild-type EC from the TCGA cohort. **(G)** Kaplan–Meier survival curves for patients with LATS1 or LAST2 mutated, LATS1/2 mutated, and LATS1/2 wild-type EC in the TCGA EC cohort. **(H, I)** Relative mRNA expression of LATS1 **(H)** or LATS2 **(I)** in normal endometrial tissues and EC tissues from the TCGA cohort. **(J, K)** IHC analysis of LATS1 in normal endometrial and EC tissues from our sample cohort **(J)**. Scale bar, 50 μm. Quantitative data for LATS1 protein staining in **(K)**. **(L, M)** IHC analysis of LATS2 in normal endometrial and EC tissues from our sample cohort **(L)**. Scale bar, 50 μm. Quantitative data for LATS2 protein staining in **(M)**. **(N, O)** LATS1 **(N)** or LATS2 **(O)** protein expression in EC at different FIGO stages from our sample cohort. *P* values are calculated using the Student’s t-test in **(H, I)**, the Mann-Whitney test in **(K, M)** and the Kruskal-Wallis test in **(N, O)**. *p < 0.05, **p < 0.01, ***p < 0.001

Despite the well-established tumor-suppressive functions of LATS1/2, a comprehensive exploration of genetic alterations in human cancers is currently lacking. To address this, we examined the mutation and copy-number variation (CNV) status of LATS1/2 genes using the TCGA cancer dataset from the cBioPortal database (www.cbioportal.org). Among all the examined tumor types, LATS1/2 genes exhibited the highest prevalence of mutations and genomic deletions in EC (Fig. S1A, B). We retrieved a total of 528 EC cases matched with whole-exome sequencing (WXS) data and transcriptome profiles (RNA-Seq), as well as clinical information. Of these cases, approximately 8.7% had LATS1 mutations (including 17 truncating and 43 missense mutations), whereas approximately 7.8% had LATS2 mutations (including 5 truncating and 50 missense mutations) (Fig. 1A, B). The high frequency of truncating mutations in the LATS1/2 genes in EC suggests that these mutations most likely result in a loss of function. Interestingly, the co-occurrence of LATS1 and LATS2 mutations was observed in the TCGA EC cohort (Fig. 1C, D).

In terms of the potential prognostic value, patients with LATS1 mutations tended to have a better prognosis than those with wild-type LATS1 gene (*p* = 0.0119) (Fig. 1E). LATS2 mutations also tended to have a better prognosis, although this trend was not statistically significant (*p* = 0.127) (Fig. 1F). Further grouping the patients based on their mutation status of LATS1/2 highlighted that only patients with LATS1 mutation had the most favorable prognosis (Fig. 1G). The analysis of RNA-Seq data revealed significantly lower levels of LATS1/2 transcripts in EC specimens than in normal tissues (*p* < 0.001 and *p* < 0.0001, respectively) (Fig. 1H, I). Proteomic data from the Clinical Proteomic Tumor Analysis Consortium further confirmed the lower expression of LATS1 protein in EC specimens (*p* = 0.0278) (Fig. S1C). No proteomic data were available for LATS2. Moreover, the mRNA expression of LATS1 or LATS2 was not correlated with the prognosis of EC patients (Fig. S1D, E).

The presence of LATS1/2 mutations in EC was validated using the targeted sequencing data from our own cohort. A total of 9 LATS1 mutations occurred in approximately 9.1% (9/99) of the tumor specimens, including 3 truncating mutations, 8 missense mutations. A total of 6 LATS2 mutations occurred in approximately 6.1% (6/99) of the tumor specimens, including 1 truncating mutation, and 8 missense mutations (Fig. S1F, G). The frequency of LATS1/2 mutations in our cohort was largely consistent with that observed in the TCGA cohort. Following validation of the antibody specificity for immunohistochemistry (IHC) analysis in parental and LATS1/2 double KO KLE cells (Fig. S1H), IHC analysis were performed and showed a significant downregulation of LATS1/2 protein expression in EC specimens compared with normal endometrial tissues (Fig. 1J-M). Moreover, a negative correlation was observed between LATS1 or LATS2 protein expression and the advanced clinical stage, but not the histologic grade (Fig. 1N, O, Fig. S1J, K). Collectively, these results indicate an abnormal reduction in the expression of LATS1/2 in EC and highlight the prognostic significance of their mutation status.

### LATS1/2 loss suppresses MHC-I expression in EC cells independent of the Hippo pathway

**Fig. 2:**
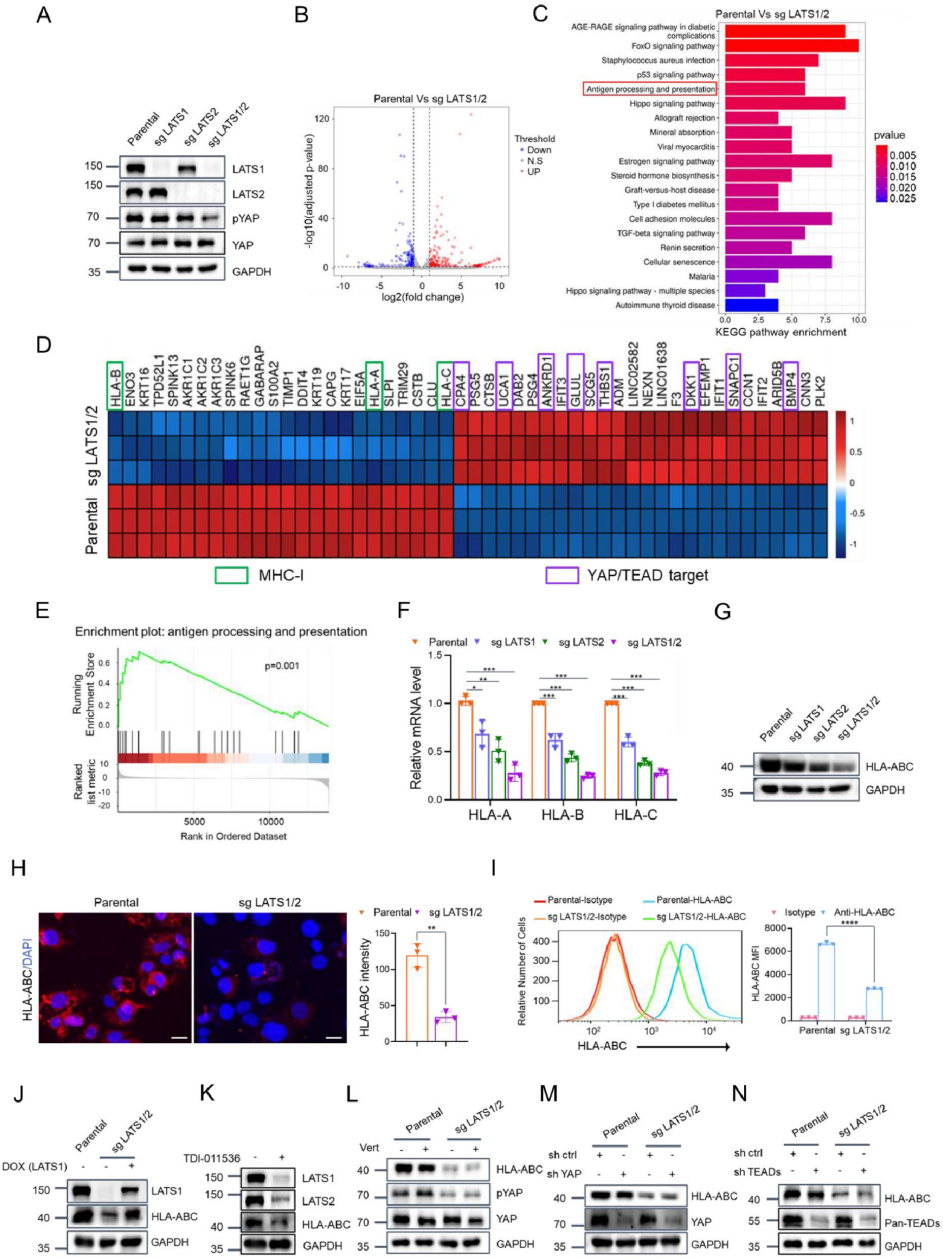
LATS1/2 loss leads to MHC-I downregulation in EC cells independent of the Hippo-YAP pathway. **(A)** Western blotting of the indicated proteins in whole cell lysates (WCLs) from KLE cells with LATS1/2 single or double KO by CRISPR-Cas9 methods. Parental KLE cells were used as a control. **(B)** Volcano plot of the differentially expressed genes in parental and LATS1/2-KO KLE cells. **(C)** KEGG pathway analysis of the differentially expressed genes in parental and LATS1/2-KO KLE cells. **(D)** Heatmap depicting the expression of the top 50 differentially expressed genes in parental and LATS1/2-KO KLE cells. **(E)** GSEA of the antigen processing and presentation gene signature in parental and LATS1/2-KO KLE cells. The hallmark antigen processing and presentation gene set (Standard name: JECHLINGER_EPITHELIAL_TO_MESENCHYMAL_TRANSITION _UP) was obtained from the Molecular Signatures Database (MsigDB).**(F)** RT-qPCR measurement of the mRNA expression of HLA-ABC in parental, LATS1-KO, LATS2-KO and LATS1/2-KO KLE cells. Data are shown as means ± SD (*n* = 3). **(G)** Western blotting of the indicated proteins in the WCLs from parental, LATS1-KO, LATS2-KO or LATS1/2-KO KLE cells. **(H)** Representative IF images of parental and LATS1/2-KO KLE cells stained with HLA-ABC (red) and DAPI (blue). Quantification of MHC-I intensity is shown on the right. Scale bar, 20 μm. Data are shown as means ± SD (*n* = 3).**(I)** MHC-I surface expression on Parental and LATS1/2-KO KLE cells detected with flow cytometry. Flow cytometric analysis was performed to determine the MFI of MHC-I. Data are shown as means ± SD (*n* = 3). **(J)** Exogenous LATS1 was reintroduced into LATS1/2-KO KLE cells to generate LATS1 Tet-On inducible cells. The cell lines were treated with (DMSO) or doxycycline (DOX) (10 ng/ml) for 24 h, and the WCLs were prepared for Western blotting. Parental KLE cells were used as the control. **(K)** Western blotting of the indicated proteins in the WCLs from KLE cells treated with TDI-011536 (3 µM) for 24 h. **(L)** Western blotting of the indicated proteins in the WCLs from parental and LATS1/2-KO KLE cells treated with DMSO or verteporfin (0.5 µM) for 24 h. **(M, N)** Western blotting of the indicated proteins in the WCLs from parental and LATS1/2-KO KLE cells stably overexpressing sh ctrl, sh YAP **(M)** or sh ctrl, sh TEADs **(N)**. P values are calculated using the Multiple t-tests in **(F, I)** and Student’s t test in **(H)**. *p < 0.05, **p < 0.01, ***p < 0.001, ****p < 0.0001

To study the biological effects of LATS1/2 loss in EC, we utilized CRISPR/Cas9 methods to create LATS1/2 single and double KO EC cell lines. The protein expression of LATS1/2 was completely eliminated in the KLE and HEC-1 A cell clones (Fig. 2A, Fig. S3A). Sanger sequencing of the genomic DNA confirmed the absence or insertion of bases in gene exons (Fig. S2A, B). Consistent with expectations, the levels of phospho-YAP(S127) were significantly decreased, indicating that LATS1/2 are essential kinases for YAP(S127) phosphorylation (Fig. 2A, Fig. S3A). RNA-Seq analysis was then conducted to identify the differentially expressed genes between parental and LATS1/2-KO KLE cells. A total of 414 protein-coding genes demonstrated altered expression in LATS1/2-KO KLE cells compared to the control cells. Of them, 224 genes were upregulated (> 2-fold), while 190 genes were downregulated (< 2-fold) (Fig. 2B, Table. S1). To investigate the functional associations of these differentially expressed genes, we performed Kyoto Encyclopedia of Genes and Genomes (KEGG) pathway enrichment analysis to determine the most significant pathways affected in LATS1/2-KO KLE cells. In addition to the Hippo pathway, the results indicated significant alterations in multiple signaling pathways, such as antigen processing and presentation, the FoxO signaling pathway, and allograft rejection, suggesting that LATS1/2 likely plays roles in these pathways (Fig. 2C, Table S2).

In this study, our focus was on investigating the potential role of LATS1/2 in the antigen processing and presentation pathway (Fig. 2E), which is often associated with tumor immune evasion. Interestingly, we observed a significant downregulation of three subtypes of MHC-I molecules, namely HLA-A, HLA-B, and HLA-C, in the LATS1 and LATS2 KO KLE cells compared to the parental cells (Fig. 2D). These findings were further validated by RT-qPCR measurements (Fig. 2F). Western blot analysis using an anti-pan-MHC-I antibody confirmed a reduction in MHC-I expression in both LATS1 KO and LATS2 KO cells (Fig. 2G). Importantly, the impact on MHC-I expression was stronger in LATS1/2-KO cells than in single KO cells, indicating that LATS1 and LATS2 play non-redundant roles in regulating MHC-I expression (Fig. 2F, G). Immunofluorescence (IF) analysis revealed a significant decrease in MHC-I staining in LATS1/2-KO KLE cells than that in parental cells (Fig. 2H). Flow cytometry analysis revealed a significant decrease in MHC-I surface expression on LATS1/2-KO KLE cells compared to parental cells (Fig. 2I). To ensure that this effect was not caused by the off-target effects of the CRISPR/Cas9 method, we employed a Tet-on-inducible expression system to introduce exogenous LATS1 expression in LATS1/2-KO KLE cells, reaching levels similar to those in the parental cells. Notably, the downregulation of MHC-I protein in LATS1/2-KO KLE cells was largely reversed upon induction of exogenous LATS1(Fig. 2J). Additionally, inhibiting LATS1/2 kinase activity with the small-molecule inhibitor TDI-011536 led to a significant reduction in MHC-I protein expression in KLE cells (Fig. 2K). Furthermore, the impact of LATS1/2 on MHC-I expression was also observed in another EC cell line, HEC-1 A (Fig. S3B-D).

Given that LATS1/2 play a crucial role in the Hippo pathway and LATS1/2 KO leads to YAP dephosphorylation and subsequent activation, we aimed to investigate whether the alteration of MHC-I expression in LATS1/2 KO cells was dependent on the Hippo-YAP pathway. However, the treatment of LATS1/2-KO cells with verteporfin, a small-molecule inhibitor of YAP-TEAD binding, failed to reverse the change in MHC-I expression (Fig. 2L, Fig. S3E). Similarly, knocking down YAP or TEADs expression using shRNAs did not reverse the altered MHC-I expression (Fig. 2M, N). Collectively, our results indicate that LATS1/2 collaboratively regulate MHC-I expression independent of the Hippo-YAP pathway.

### Loss of LATS1/2 attenuates the transcriptional responses induced by IFN-γ

**Fig. 3:**
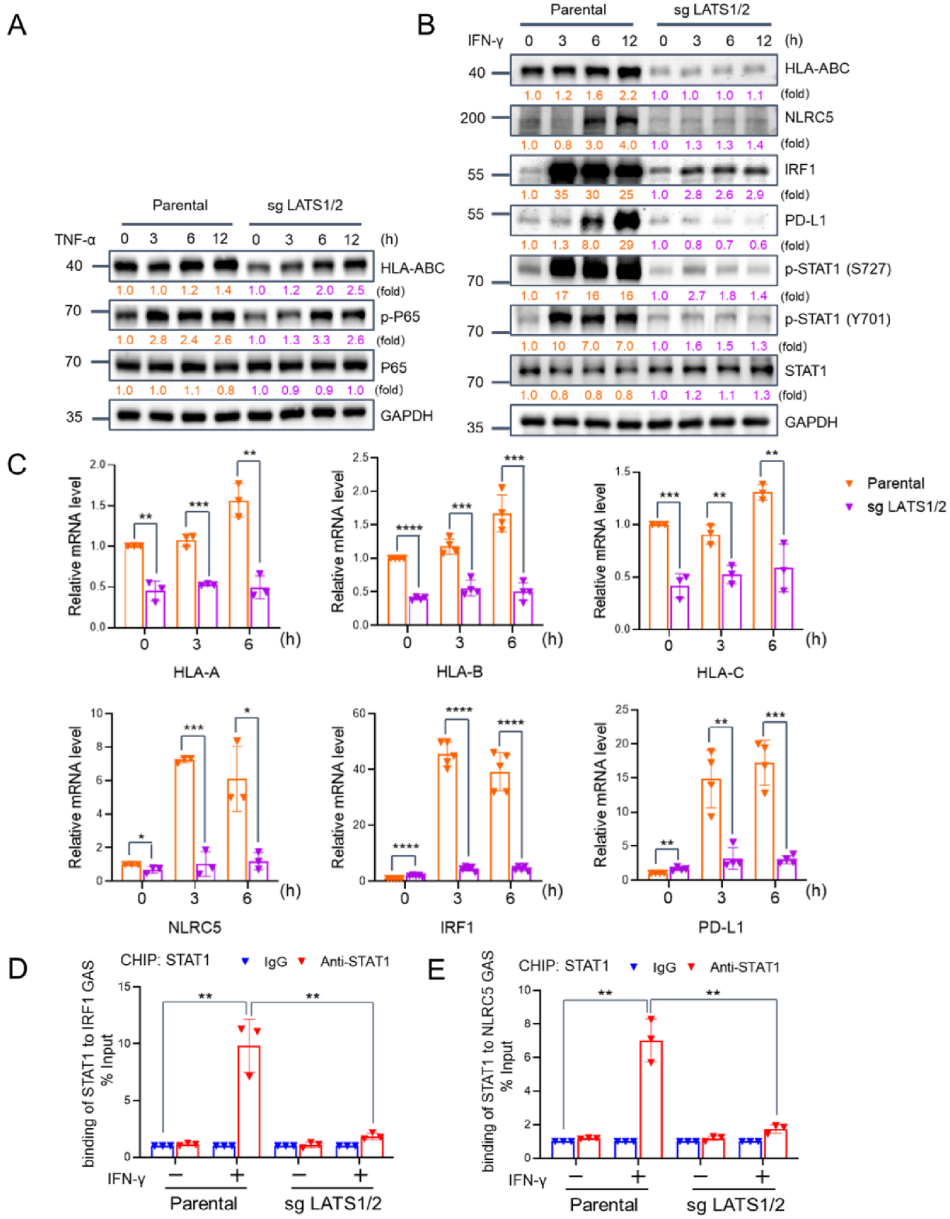
Loss of LATS1/2 attenuates the transcriptional responses induced by IFN-γ. **(A)** Western blotting of indicated proteins in the WCLs from parental and LATS1/2-KO KLE cells treated with TNFα (20 ng/ml) and harvested at different time points. **(B)** Western blotting of indicated proteins in the WCLs from parental and LATS1/2-KO KLE cells treated with IFN-γ (30 ng/ml) and harvested at different time points. **(C)** RT-qPCR measurement of the mRNA expression of IFN-γ target genes in parental and LATS1/2-KO KLE cells treated with IFN-γ (30 ng/ml) and harvested at different time points. Data are shown as means ± SD (*n* = 3). **(D, E)** ChIP-qPCR measurement of the enrichment of STAT1 in NLRC5 **(D)** and IRF1 **(E)** promoter regions treated with IFN-γ (30 ng/ml) for 6 h. *P* values are calculated using the Multiple t-tests in **(C)** and One-way ANOVA test in **(D, E)**. *p < 0.05, **p < 0.01, ***p < 0.001, ****p < 0.0001

Previous studies have identified three distinct transcription binding sites that regulate MHC-I heavy chain expression, namely Enhancer A (recognized by NF-κB), ISRE site (recognized by IRF1), and SXY-module (recognized by NLRC5) [6]. IRF1 and NLRC5 are two transcription factors induced by IFN-γ; therefore, we investigated whether LATS1/2 loss suppresses the transcriptional activation of MHC-I through these pathways. LATS1/2-KO KLE cells were treated with TNF-α to trigger NF-κB activation. Phosphorylation and subsequent nuclear translocation of P65 plays a vital role in TNF-α-induced NF-κB activation. As shown in Fig. 3A, the loss of LATS1/2 did not attenuate the transcriptional activation of MHC-I induced by TNF-α. TNF-α-induced P65 phosphorylation and nuclear localization of P65 was also not compromised in LATS1/2-KO KLE cells compared to parental cells (Fig. 3A, Fig. S4A, B). These results further reinforced the notion that LATS1/2 is not involved in NF-κB-mediated transcriptional activation of MHC-I induced by TNF-α. On the other hand, IFN-γ treatment-induced MHC-I protein and mRNA expression was significantly diminished in LATS1/2-KO KLE cells. Moreover, the protein and mRNA expression levels of IRF1, NLRC5, and PD-L1, all of which are transcriptional targets of STAT1 and induced by IFN-γ treatment, were also markedly impaired in LATS1/2-KO KLE cells (Fig. 3B, C). Furthermore, the impact of LATS1/2 on IFN-γ-induced transcriptional outputs was also observed in another EC cell line, SPEC-2, with or without TDI-011536 treatment (Fig. S4D-E).

Upon IFN-γ treatment, the phosphorylation of STAT1 at Y701 and S727 is essential for its nuclear translocation and activation. However, we observed a significant reduction in STAT1 phosphorylation at these sites in LATS1/2-KO KLE cells, indicating that LATS1/2 plays a regulatory role upstream of STAT1. Additionally, the chromatin immunoprecipitation (ChIP)-qPCR assay results revealed decreased STAT1 enrichment in the promoter regions of NLRC5 and IRF1 (which are stimulated by IFN-γ) in LATS1/2-KO KLE cells compared to parental cells (Fig. 3D, E). Collectively, these results indicate that the loss of LATS1/2 hinders IFN-γ-induced transcriptional outputs, potentially by inhibiting STAT1 activation.

### LATS1/2 directly interact with STAT1 and catalyze STAT1 phosphorylation

**Fig. 4:**
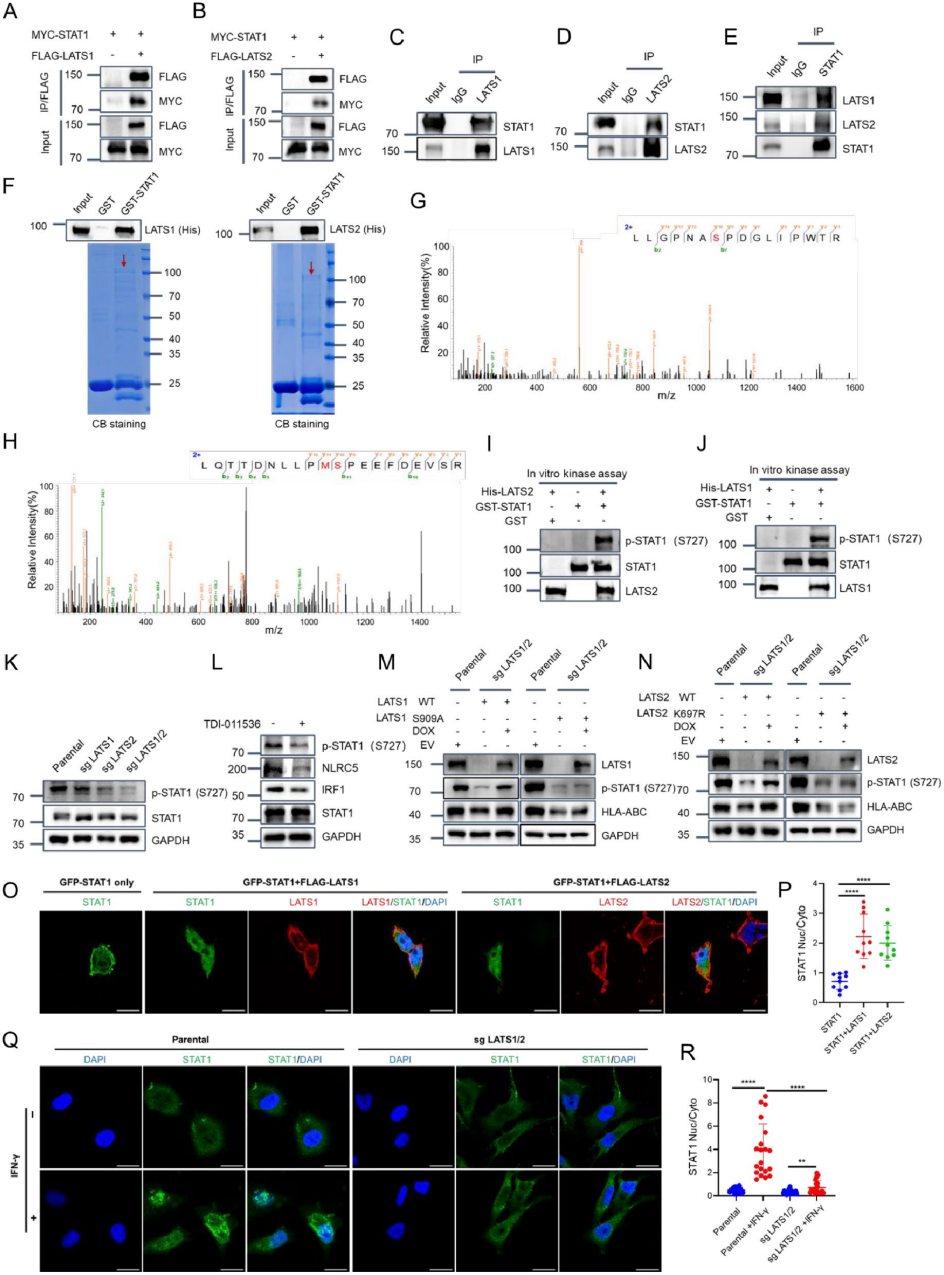
LATS1/2 directly interacts with STAT1 and phosphorylates STAT1. **(A, B)** Western blotting of the WCLs and co-IP samples of anti-FLAG antibody obtained from 293T cells transfected with the indicated plasmids. **(C-E)** Immunoprecipitation using anti-LATS1 **(C)**, anti-LATS2 **(D)**, or anti-STAT1 **(E)** antibodies in the WCLs prepared from KLE cells followed by western blotting with the indicated antibodies. **(F)** Recombinant expressed GST-STAT1 protein or GST bound to glutathione-Sepharose beads and incubated with recombinant expressed His-LATS1 or LATS2 proteins. Bound His-LATS1 or LATS2 proteins were detected by western blotting with anti-His antibody. **(G, H)** Recombinant GST-STAT1_1 − 650aa_ or GST-STAT1_650 − 750aa_ proteins were subjected to in vitro phosphorylation by active human His-LATS2_480 − 1088aa_ protein. The MS spectra correspond to two phosphorylated STAT1 peptides are shown. **(I)** Recombinant GST-STAT1 proteins were subjected to phosphorylation by active human His-LATS2_480 − 1088aa_ protein, as detected using in vitro kinase assays. The reaction products were subjected to western blotting. **(J)** Recombinant GST-STAT1 proteins were subjected to phosphorylation by active human His-LATS1_589 − 1130aa_ protein, as detected using in vitro kinase assays. The reaction products were subjected to western blotting. **(K)** Western blotting of the indicated proteins in the WCLs from parental, LATS1-KO, LATS2-KO or LATS1/2-KO KLE cells. **(L)** Western blotting of the indicated proteins in WCLs from KLE cells treated with TDI-011536 (3 µM) for 24 h. **(M)** Exogenous LATS1 (WT or kinase dead mutant) was reintroduced into LATS1/2-KO KLE cells to generate LATS1 Tet-On inducible cells. The cell lines were treated with DMSO or doxycycline (DOX) (10 ng/ml) for 24 h, and the WCLs were prepared for western blotting. **(N)** Exogenous LATS2 (WT or kinase dead mutant) was reintroduced into LATS1/2-KO KLE cells to generate LATS2 Tet-On inducible cells. The cell lines were treated with DMSO or DOX (10 ng/ml) for 24 h, and the WCLs were prepared for western blotting. **(O, P)** Representative IF images from 293T cells transfected with the indicated plasmids and stained with LATS1 (FLAG), LATS2 (FLAG), STAT1 (pEGFP) and DAPI **(O)**. Scale bar, 20 μm. Quantification of the ratio of STAT1 in Nuc (nucleus)/Cyto (cytoplasm) is shown in **(P)**. Data are shown as means ± SD (*n* = 10). **(Q, R)** Representative IF images from parental and LATS1/2-KO KLE cells treated with IFN-γ (30 ng/ml) for 6 h, stained with STAT1 and DAPI **(Q)**. Scale bar, 20 μm. Quantification of the ratio of STAT1 in Nuc/Cyto is shown in **(R)**. Data are shown as means ± SD (*n* = 20). *P* values are calculated using One-way ANOVA test in **(P, R)**. *p < 0.05, ****p < 0.0001

We then explored the underlying mechanisms by which LATS1/2 inhibit the transcriptional activity of STAT1. Indeed, exogenous and endogenous co-IP assay results indicated that LATS1/2 can interact with STAT1 in cells (Fig. 4A-E). To determine the direct interaction between LATS1/2 and STAT1, we conducted in vitro experiments using purified recombinant proteins. Specifically, we expressed and purified recombinant GST-STAT1 and His-LATS1/2 in bacteria. As shown in Fig. 4F, GST-STAT1 protein, but not GST alone, exhibited binding to His-LATS1 or LATS2, as indicated by the results of GST pull-down assays. This confirmed the physical interaction between STAT1 and LATS1/2. Given that LATS1/2 are serine/threonine kinases, we investigated whether they could directly phosphorylate STAT1 by performing in vitro kinase assays using recombinant active LATS2 and either GST-STAT_1 − 650aa_ or the STAT1_650 − 750aa_ segment as substrates. Mass spectrometry analysis of phospho-peptides revealed that LATS2 phosphorylate residues S532 and S727 in STAT1 (Fig. 4G, H). Previous literature has not documented the S532 phosphorylation of STAT1. However, it has been reported that the S727 phosphorylation of STAT1 is crucial for its activation in response to IFN signaling. In our study, we utilized an anti-phospho-STAT1(S727) antibody and demonstrated that LATS1 and LATS2 active proteins could phosphorylate STAT1 at S727 (Fig. 4I, J). We observed a marked downregulation of STAT1 S727 phosphorylation in LATS1/2-KO KLE and HEC-1 A cells (Fig. 4K, Fig. S5A). Additionally, treatment with the LATS1/2 inhibitor TDI-011536 resulted in a notable decrease in phospho-STAT1(S727), IRF1, and NLRC5 expression in KLE and HEC-1 A cells (Fig. 4L, Fig. S5B). The downregulation of phospho-STAT1(S727) and MHC-I in LATS1/2-KO KLE cells was reversed upon the reintroduction of wild-type LATS1 or LATS2 but not their kinase-dead mutant (LATS1-S909A, LATS2-K697R) (Fig. 4M, N, Fig. S5C, D). Nuclear translocation plays a vital role in IFN-γ-induced STAT1 activation. Co-expression of LATS1 or LATS2 with STAT1 in 293T cells resulted in the nuclear localization of STAT1 (Fig. 4O, P). Conversely, IFN-γ-induced nuclear localization of STAT1 was significantly compromised in LATS1/2-KO KLE cells than in parental cells (Fig. 4Q, R). Collectively, these results indicate that LATS1/2 directly interact with STAT1, promoting STAT1 phosphorylation and nuclear localization.

### LATS1/2 loss promotes tumor immune evasion by downregulating MHC-I expression

**Fig. 5:**
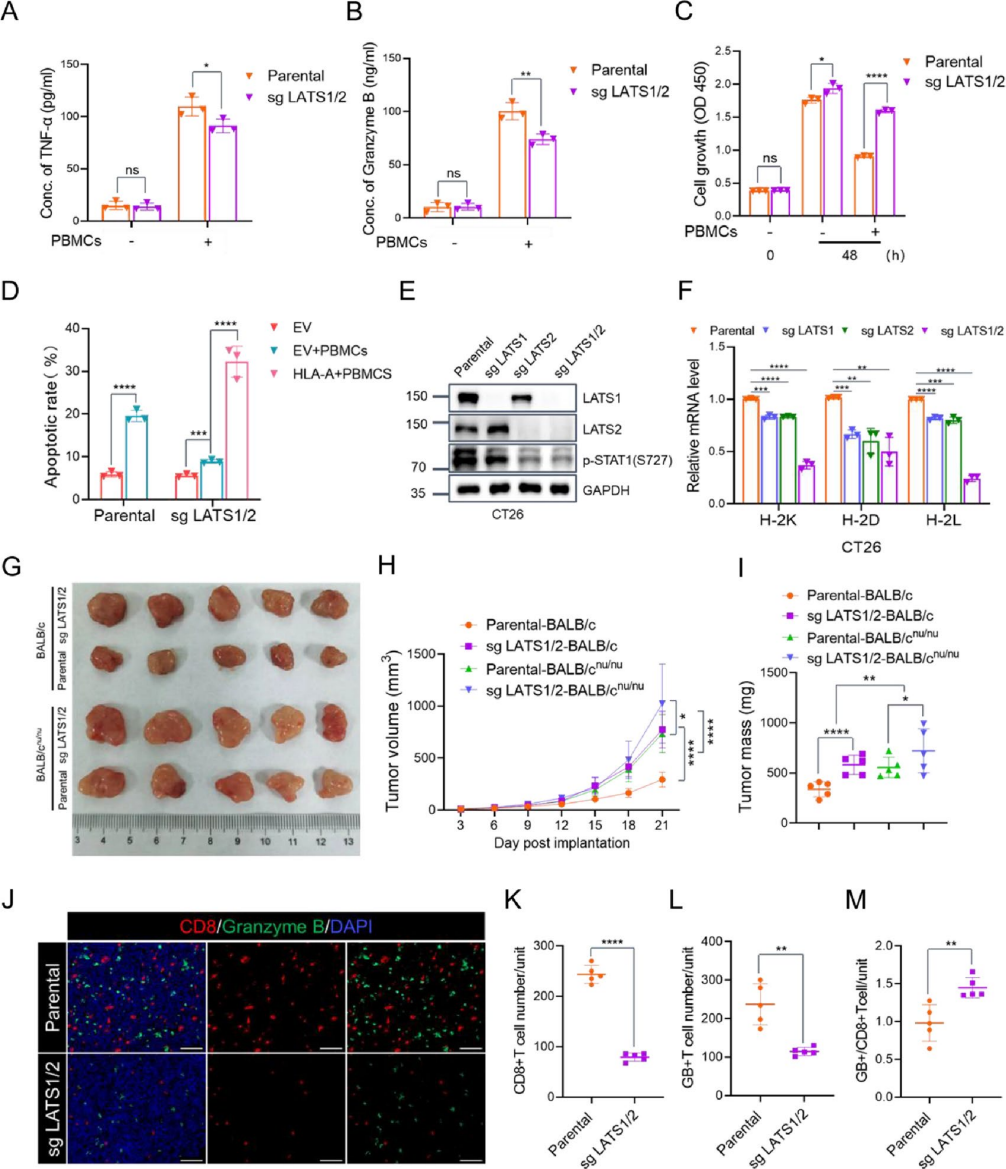
LATS1/2 loss promotes tumor immune evasion by downregulating MHC-I expression. **(A)** The concentration of TNF-α in the supernatant of PBMCs and EC cells co-culture system. Data are shown as means ± SD (*n* = 3). **(B)** The concentration of Granzyme B in the supernatant of PBMCs and EC cells co-culture system. Data are shown as means ± SD (*n* = 3). **(C)** CCK-8 cell proliferation analysis of parental and LATS1/2-KO KLE cells co-cultured with PBMCs for 48 h. Data are shown as means ± SD (*n* = 3). **(D)** Parental and LATS1/2-KO KLE cells or exogenous HLA-A reconstituted LATS1/2-KO KLE cells were co-cultured with PBMCs for 24 h. Flow cytometric analysis was performed to determine the apoptotic rates of KLE cells. Data are shown as means ± SD (*n* = 3). **(E)** Western blotting of the indicated proteins in the WCLs from parental, LATS1-KO, LATS2-KO or LATS1/2-KO CT26 cells. **(F)** RT-qPCR measurement of the mRNA expression of mouse MHC-I in parental, LATS1-KO, LATS2-KO or LATS1/2-KO CT26 cells. Data are shown as means ± SD (*n* = 3). **(G-I)** Parental or LATS1/2-KO CT26 cells were injected subcutaneously into the flank of BALB/c or BALB/c^nu/nu^ mice. Tumor growth was measured every 3 days for 21 days. 5 mice per experimental group. Tumors in each group at day 21 were harvested and photographed **(G)**, tumor volume **(H)** and tumor weight **(I)** at each time point was documented. Data are shown as means ± SD (*n* = 5). **(J-M)** Immunostaining of CD8 and Granzyme B in the CT26 tumor mass in BALB/c mice **(J)**. DAPI: nuclear counterstaining **(J)**. Scale bar, 100 μm. The intensity of CD8 **(K)** and Granzyme B **(L)** were quantified using Image J. The ratio of Granzyme B^+^/CD8^+^ T cells was shown in **(M)**. Data are shown as means ± SD (*n* = 5). 1 tissue slide per tumor, 5 mice per group. Unit = 1.9 × 10^6^ µm^2^. *P* values are calculated using the Multiple t-tests in **(A, B, C, D and F)**, Two-way ANOVA test in **(H)**, One-way ANOVA test in **(I)** and Student’s t test in **(K, L and M)**. *p < 0.05, **p < 0.01, ***p < 0.001, ****p < 0.0001, ns: no significant

MHC-I plays a crucial role in mediating anti-tumor immunity and CD8^+^ T cell cytotoxicity [7]. The profound decrease in MHC-I expression observed in LATS1/2 KO cells suggests a potential weakening of anti-tumor immunity. To test this hypothesis, we isolated peripheral blood mononuclear cells (PBMCs) from healthy female volunteers and activated them using PHA. These activated PBMCs were then co-cultured with EC cells. The secretion of TNF-α or Granzyme B in the supernatant was measured to assess the killing activity of immune cells. As shown in Fig. 5A, B, the levels of TNF-α or Granzyme B in LATS1/2 KO cells co-culture system was significantly lower than that in parental cells co-culture system. Then, the viability of EC cells in co-culture system was determined by CCK-8 cell proliferation assays, and the results showed that LATS1/2 loss strongly attenuated the killing effect of activated PBMCs on KLE and HEC-1 A cells (Fig. 5C, Fig. S6A). Notably, co-culture cytotoxicity assay by cell apoptosis analysis with flow cytometry confirmed that the stable overexpression of HLA-A in LATS1/2-KO KLE cells reversed the resistance to activated PBMC-mediated cytotoxicity induced by LATS1/2 loss (Fig. 5D, Fig. S6B, C). While reducing the surface expression of MHC-I enables evasion from T-cell-mediated anti-tumor immunity, low MHC-I expression renders cells susceptible to cytotoxicity by Natural Killer (NK) cells [25].

Therefore, cell apoptosis analysis using flow cytometry was conducted to determine NK cell-mediated cytotoxicity. As shown in Fig. S6D, E, the loss of LATS1/2 increased the susceptibility of KLE cells to NK cell cytotoxicity. Moreover, this effect was reversed by stable overexpression of HLA-A.

We postulated that LATS1/2 loss reduces MHC-I expression and impairs MHC-I-mediated antigen presentation in tumor cells, leading to a decrease in CD8^+^ T cells infiltration in allograft tumors. However, to the best of our knowledge, no murine-derived EC cell lines have been generated and documented in the previous literature. To validate this hypothesis, we conducted a subcutaneous tumor transplantation experiment in BALB/c mice using the CT26 murine colon carcinoma model, commonly used to study tumor immune evasion in immunocompetent mouse models. We generated LATS1/2 single and double KO CT26 cells using CRISPR/Cas9 methods (Fig. 5E, Fig. S2C). Consistent with the observation in human EC cell lines, the levels of phospho-STAT1(S727) were significantly reduced in LATS1/2 KO CT26 cells (Fig. 5E). Although there is no commercial antibody available for human MHC-I homologs in mice, we assessed changes in the mRNA levels of mouse MHC-I using RT-qPCR. Our results showed a significant downregulation of three subtypes of mouse MHC-I molecules (H-2 K, H-2D, and H-2 L) in LATS1/2 KO CT26 cells compared to parental cells (Fig. 5F). Furthermore, we performed subcutaneous transplantation of parental and LATS1/2-KO CT26 cells into immunodeficient BALB/c^nu/nu^ mice as well as immunocompetent BALB/c mice. The results showed that LATS1/2 loss accelerated tumor growth more significantly in immunocompetent mice than in immunodeficient mice, highlighting the critical role of the host immune system in LATS1/2-mediated anti-tumor effect (Fig. 5G-I). Given that CD8 + cytotoxic T lymphocytes (CTLs) eliminate cancer cells by secreting Granzyme B (GB), a potent inducer of tumor cell apoptosis, we investigated the CD8 + CTL population and CTL activity by measuring GB release in xenograft CT26 tumors using immunofluorescence analysis. As showed in Fig. 5J-M, the loss of LATS1/2 significantly reduced both the CD8 + CTL population and GB release in CT26 tumors. Collectively, these results indicate that LATS1/2 loss impairs the infiltration of CD8^+^ T cells in tumors and may hinder anti-tumor immunity by suppressing MHC-I expression.

### LATS1/2 expression is positively correlated with MHC-I in EC

**Fig. 6:**
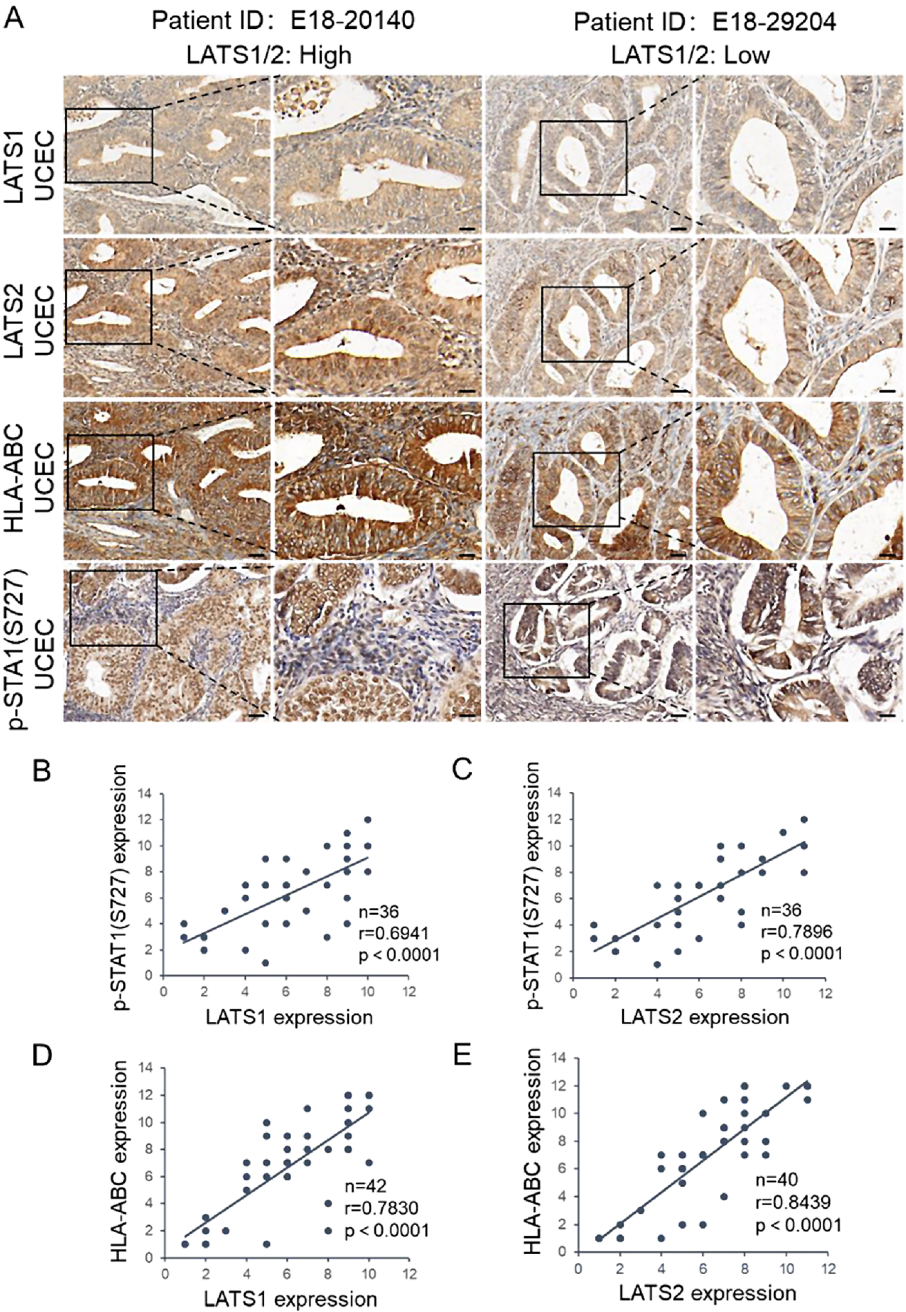
LATS1/2 expression are positively correlated with MHC-I and phospho-STAT1 (S727) in EC. **(A)** IHC analysis of LATS1, LATS2, HLA-ABC, and p-STAT1 (S727) in 50 primary EC specimens. Scale bar, 50 μm. The inset in each panel shows a high magnification image of the representative (framed) area. Scale bar, 20 μm. **(B, C)** Correlation analysis of phospho-STAT1 (S727) IHC intensity with LATS1 **(B)** and LATS2 **(C)**. **(D, E)** Correlation analysis of MHC-I IHC intensity with LATS1 **(D)** and LATS2 **(E)**. *P* values are calculated using the Correlation **(B-E)**

The above results have demonstrated that LATS1/2 loss leads to the downregulation of phospho-STAT1(S727) and MHC-I in EC cells. To further explore the pathological relevance of LATS1/2, MHC-I, and phospho-STAT1(S727) in EC, we performed IHC staining of 42 primary EC specimens (Fig. 6A). The results revealed a positive correlation between the intensity of LATS1/2 staining and phospho-STAT1 (S727) levels (Fig. 6B, C). Moreover, we observed a positive correlation between LATS1/2 intensity and MHC-I expression (Fig. 6D, E). These findings suggest that the downregulation of LATS1/2 is associated with the aberrant expression of phospho-STAT1(S727) and MHC-I, potentially implicating a pathological role for LATS1/2 in EC tumorigenesis.

## Discussion

**Fig. 7:**
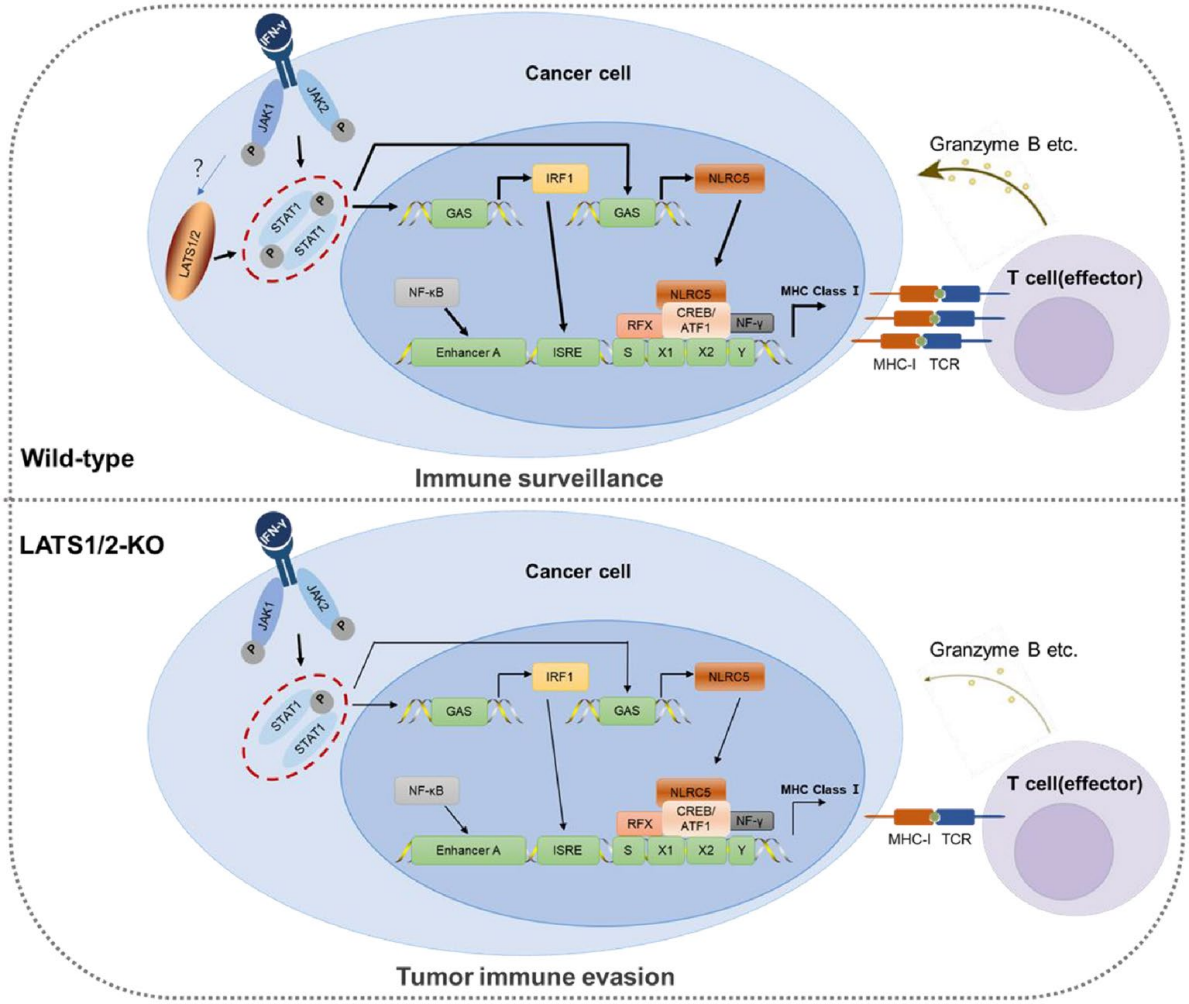
Schematic diagram depicts the function that LATS1/2 upregulates MHC-I expression through positively modulating the IFN-γ-STAT1-IRF1 signaling. LATS1/2 directly interact with and phosphorylate STAT1 at Ser727, a crucial transcription factor for MHC-I upregulation in response to interferon-gamma (IFN-γ) signaling, to promote STAT1 accumulating and moving into the nucleus to enhance the transcriptional activation of IRF1/NLRC5 on MHC-I. The dysregulation of LATS1/2 in EC leads to immune evasion through the down-regulation of MHC-I, which may result in primary and acquired resistance to immune checkpoint blockade therapy

The deregulation of MHC-I molecules plays a vital role in tumor immune evasion. Understanding the molecular basis of MHC-I deregulation in EC is crucial for developing effective immunotherapeutic strategies to restore immune recognition and enhance anti-tumor responses [7]. In EC, various mechanisms lead to the diminished expression of MHC-I molecules in tumor cells. They include decreased expression of MHC-I genes, abnormal post-translational modifications, and interference with the peptide-loading machinery. Consequently, tumor cells become less visible to the immune system, avoiding detection and destruction by cytotoxic T cells. Bijen et al. reported that downregulated MHC-I has a negative impact on disease-specific survival, observed in a large cohort of patients with EC [26]. Jong et al. reported that loss of MMR protein expression is related to the selective downregulation of HLA class I, which contributes to immune escape in EC [27]. Zhan et al. conducted a study showing that the autophagy-related protein LC3 interacts with NLRC5 to impede the NLRC5-mediated MHC-I antigen presentation pathway, which suggests that targeting LC3 inhibition and promoting NLRC5 expression could be a potential immunotherapeutic strategy for the treatment of EC [28]. EC shows an exceptionally high frequency of loss-of-function mutations in JAK1, a key mediator of IFN-γ signaling, in comparison to other cancer types [29, 30]. In this study, we have introduced a new tumor immune evasion mechanism utilized by EC that involves the engagement of LATS1/2. This mechanism leads to the downregulation of MHC-I expression at the transcriptional level, as depicted in the schematic diagram (Fig. 7). This decrease in MHC-I expression can potentially confer primary and acquired resistance to ICB therapy in LATS1/2-mutated EC cells.

It has been documented that phosphorylation of Ser727 in STAT1 is essential for its transcriptional activity and full activation of the IFN-γ signaling pathway [16, 20, 31, 32]. Various protein kinases have been identified to mediate the phosphorylation of STAT1 at Ser727 in response to different stimuli. One such kinase is p38, a member of the mitogen-activated protein kinase (MAPK) family. Activation of p38 MAPK can lead to the phosphorylation of STAT1 at Ser727, enhancing its transcriptional activity and promoting IFN-γ signaling [33]. Protein kinase C (PKC)-δ has also been reported to phosphorylate STAT1 at Ser727. KC-δ specifically has been implicated in the regulation of immune responses and inflammation, and its activation can lead to the phosphorylation of STAT1 and subsequent enhancement of STAT1-mediated gene expression [21, 34]. Furthermore, cyclin-dependent kinase 8 (CDK8), a member of the CDK family involved in cell cycle regulation and transcriptional control, has been identified as another kinase that can phosphorylate STAT1 at Ser727. CDK8, along with its partner cyclin C, forms the Mediator complex, which interacts with transcription factors and regulates gene expression. Phosphorylation of STAT1 by CDK8 can enhance its transcriptional activity and promote IFN-γ signaling [22]. In our study, we have revealed that LATS1/2 kinases have the capability to phosphorylate the STAT1 protein at Ser532 and Ser727 residues using in vitro kinase assays. However, the lack of site-specific phosphorylation antibody prevents us from directly confirming if STAT1 Ser532 is regulated by LATS1/2 in cells. Nevertheless, data obtained from several quantitative phosphoproteomic studies available in the Phosphosite database (www.phosphosite.org/) have demonstrated the in vivo phosphorylation of this site sites. However, until now, the kinases responsible for this phosphorylation event have not been reported. In contrast, the downregulation of phospho-STAT1(Ser727) in LATS1/2-KO cells, both in basal conditions and under IFN-γ treatment, strongly suggests that this site is indeed an authentic phosphorylation site catalyzed by LATS1/2. The consensus motif for LATS1/2 phosphorylation has been identified as “HXRXX(S/T)”. This motif is present within the regulatory domains of YAP and TAZ, and its phosphorylation by LATS1/2 plays a critical role in regulating their activity and subcellular localization [35]. The protein sequence surrounding Ser727 does not match this consensus motif. However, there are non-canonical LATS-regulated events that do not conform to this consensus motif [24]. This indicates that LATS substrate selection may be influenced by factors beyond the specific amino acid sequence. Another issue that we did not clarify in the current study is how IFN-γ signaling triggers the activation of LATS1/2, which needs to be investigated in future research. Collectively, our findings, along with others, indicate that various cell types can harness multiple kinases to facilitate STAT1 Ser727 phosphorylation, thereby enhancing IFN-γ signaling.

## Conclusion

In this study, we revealed a new tumor immune evasion mechanism utilized by endometrial cancer (EC) that involves the downregulation of LATS1/2. Specifically, LATS1/2 directly interact with and phosphorylate STAT1 at Ser727, a crucial transcription factor for MHC-I upregulation in response to interferon-gamma (IFN-γ) signaling, to promote STAT1 accumulating and moving into the nucleus to enhance the expression of MHC-I, independently of the canonical Hippo-YAP pathway. Therefore, dysregulation of LATS1/2 in EC leads to immune evasion partly through the down-regulation of MHC-I, which may result in primary and acquired resistance to immune checkpoint blockade therapy. We believe that our study makes a significant contribution to the literature because we illuminate the molecular basis of MHC-I deregulation in EC, which is crucial for developing effective immunotherapeutic strategies to restore immune recognition and enhance the anti-tumor response.

### Electronic supplementary material

Below is the link to the electronic supplementary material.

Supplementary Material 1

Supplementary Material 2

## Data Availability

The datasets presented in this manuscript are available from the corresponding author on reasonable request.

## Abbreviations

EC: Endometrial cancer
MHC-I: Major histocompatibility complex class I
HLA-I: Human leucocyte antigen I
IRF1: Interferon regulatory factor 1
NLRC5: NOD-like receptor family CARD domain containing 5
YAP: Yes-associated protein
TAZ: PDZ-binding motif
KO: Knockout
IFN-γ: Interferon-gamma
NF-κB: Nuclear Factor NF-Kappa-B
TNF-α: Tumor necrosis factor-α
GB: Granzyme B
CCK8: Cell Counting Kit-8
WB: Western blot
RT-qPCR: Reverse transcription quantitative PCR
IHC: Immunohistochemistry
IF: Immunofluorescence
ChIP: Chromatin Immunoprecipitation
Co-IP: Co-immunoprecipitation
TCGA: The Cancer Genome Atlas
GEO: Gene Expression Omnibus
KEGG: Kyoto Encyclopedia of Genes and Genomes
