# Supplementary material for: LATS1/2 loss promote tumor immune evasion in endometrial cancer through downregulating MHC-I expression
Source: J Exp Clin Cancer Res. 2024 Feb 21;43:54. doi: 10.1186/s13046-024-02979-z (PMC10880206; doi:10.1186/s13046-024-02979-z)
Supplement: Supplementary file 1 — Supplementary Material 1 [file 13046_2024_2979_MOESM1_ESM.pdf]

Supplementary Materials for

LATS1/2 Loss Promote Tumor Immune Evasion in Endometrial Cancer through Downregulating MHC-I Expression

*Corresponding author: Kun Gao, kungao@tongji.edu.cn; Xiaoping Wan, wanxiaoping@tongji.edu.cn.

This PDF file includes:

Figs. S1 to S6

Tables S1 to S8: Supplemented in a separate Excel files

Table S1. The differentially expressed genes in LATS1/2-KO KLE cells.

Table S2. KEGG enrichment of differentially expressed genes in LATS1/2-KO KLE cells.

Table S3. Chemical reagents and antibodies.

Table S4. shRNA sequence information.

Table S5. Primer sequence.

Table S6. Recombinant DNA.

Table S7. IHC scores and associated clinical information of 42 ECs and 8 normal endometrial tissues.

Table S8. LATS1 and LATS2 mutation status in 99 cases of endometrial cancer specimens and the associated clinical information.

Supplementary Figures

Fig. S1

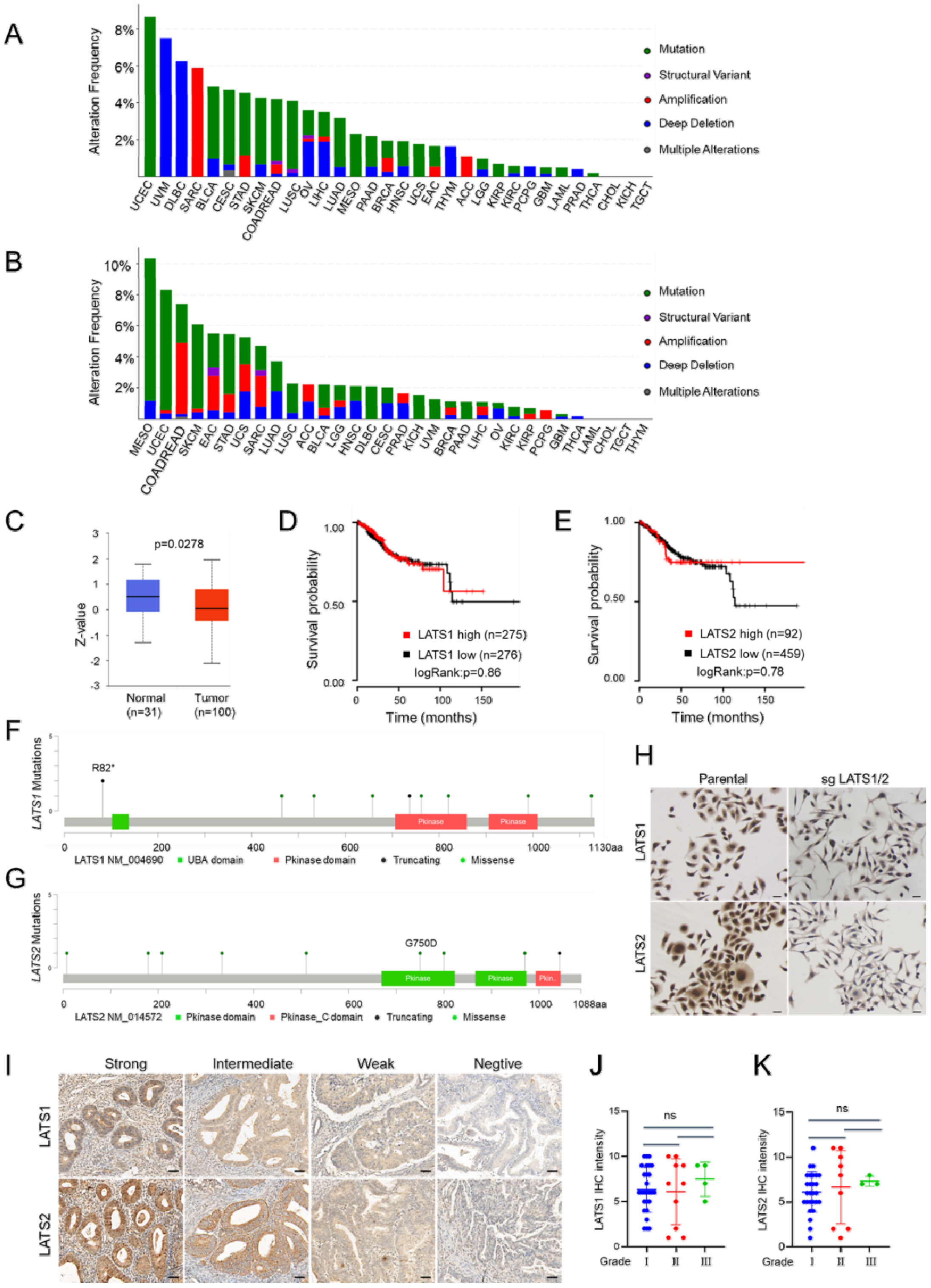

Fig. S1. LATS1 and LATS 2 are frequently mutated and downregulated in patients with EC (related to Fig. 1).

(A, B) Mutations of LATS1/2 genes among all the examined tumor types.

(C) Relative protein expression of LATS1 in normal endometrial and endometrial cancer tissues from the CPTAC EC cohort.

(D,E) Kaplan–Meier survival curves for patients with high vs. low mRNA expression of LATS1 (D) or LATS2 (E) from the TCGA EC cohort.

(F,G) Schematics of the LATS1 (F) and LATS2 (G) proteins showing the positions of individual somatic mutations identified in our sample cohor (99 cases primary ECs).

(H) Representative images of LATS1 and LATS2 ICC staining in parental and LATS1/2-KO KLE cells. Scale bar, 50 μm.

(I) Representative images of LATS1 and LATS2 IHC staining in EC tissues (strong, intermediate, weak, negative). Scale bar, 50 μm.

(J,K) Relationship between LATS1 (J) or LATS2 (K) protein expression and histologic grade from our sample cohort.

P values are calculated using the Student's t-test in (C) and the Kruskal-Wallis test in (J, K).

Fig. S2

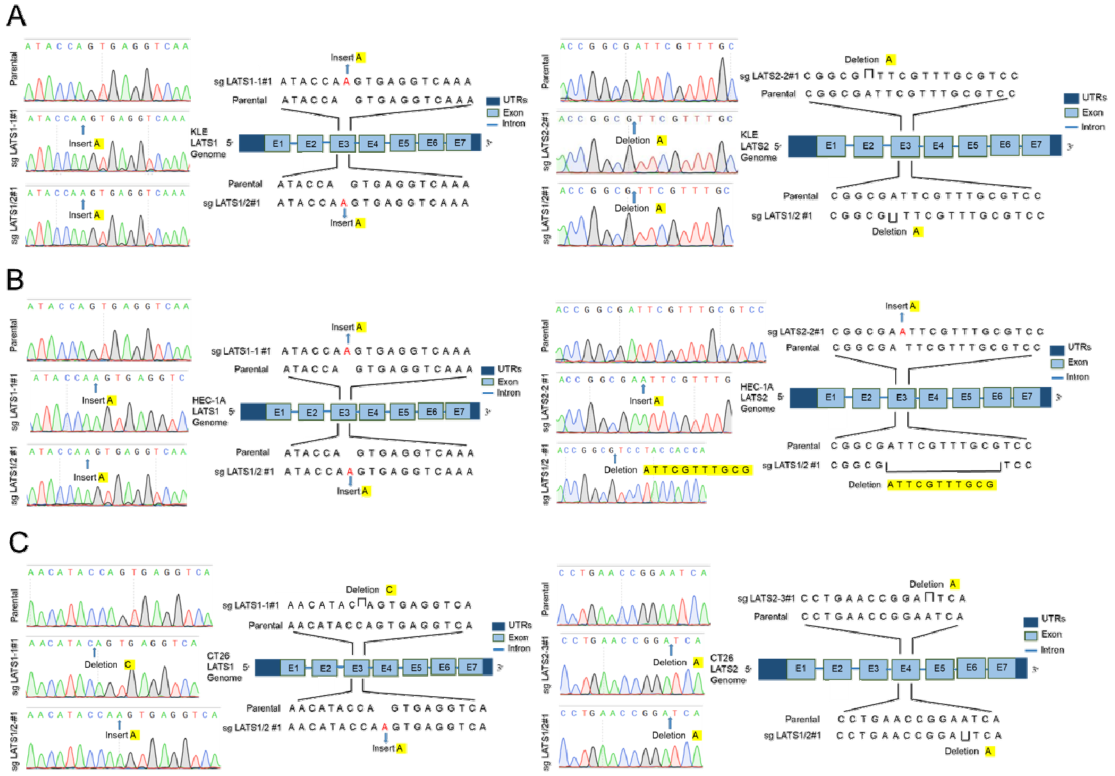

Fig. S2. Validation of LATS1/2 KO in cell lines.

(A-C) Schem Sanger sequencing confirming that LATS1 and LATS2 gene were successfully edited in KLE (A), HEC-1A (B) and CT26 (C) cells.

Fig. S3

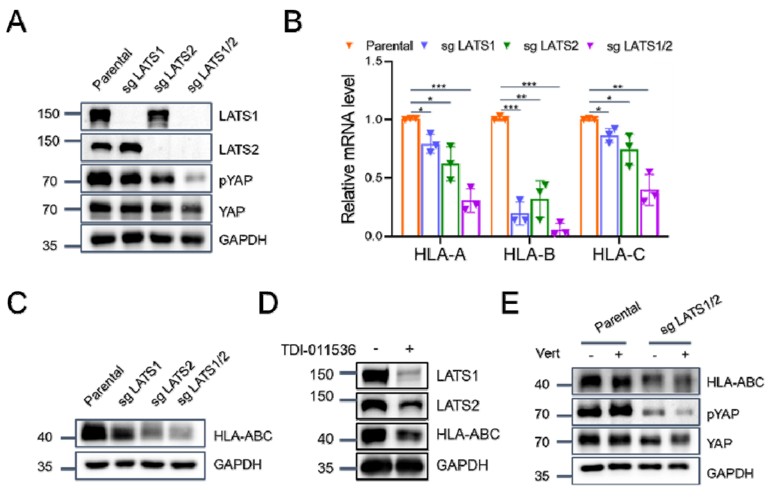

Fig. S3. LATS1/2 loss leads to MHC-I downregulation in EC cells independent of the Hippo-YAP pathway (related to Fig. 2).

(A) Western blotting of the indicated proteins in the WCLs from parental, LATS1-KO, LATS2-KO or LATS1/2-KO HEC-1A cells.

(B) RT-qPCR measurement of the mRNA expression of HLA-ABC in parental, LATS1-KO, LATS2-KO and LATS1/2-KO HEC-1A cells. Data are shown as means ± SD (n=3).

(C) Western blotting of the indicated proteins in the WCLs from parental, LATS1-KO, LATS2-KO and LATS1/2-KO HEC-1A cells.

(D) Western blotting of the indicated proteins in WCLs from HEC-1A parental cells treated with TDI-011536 (3 μM) for 24 h.

(E) Western blotting of the indicated proteins in WCLs from parental and LATS1/2-KO HEC-1A cell streated with DMSO or verteporfin (0.5 μM) for 24 h.

P values are calculated using the Multiple t-tests in (B). *p < 0.05, **p < 0.01, ***p < 0.001.

Fig. S4

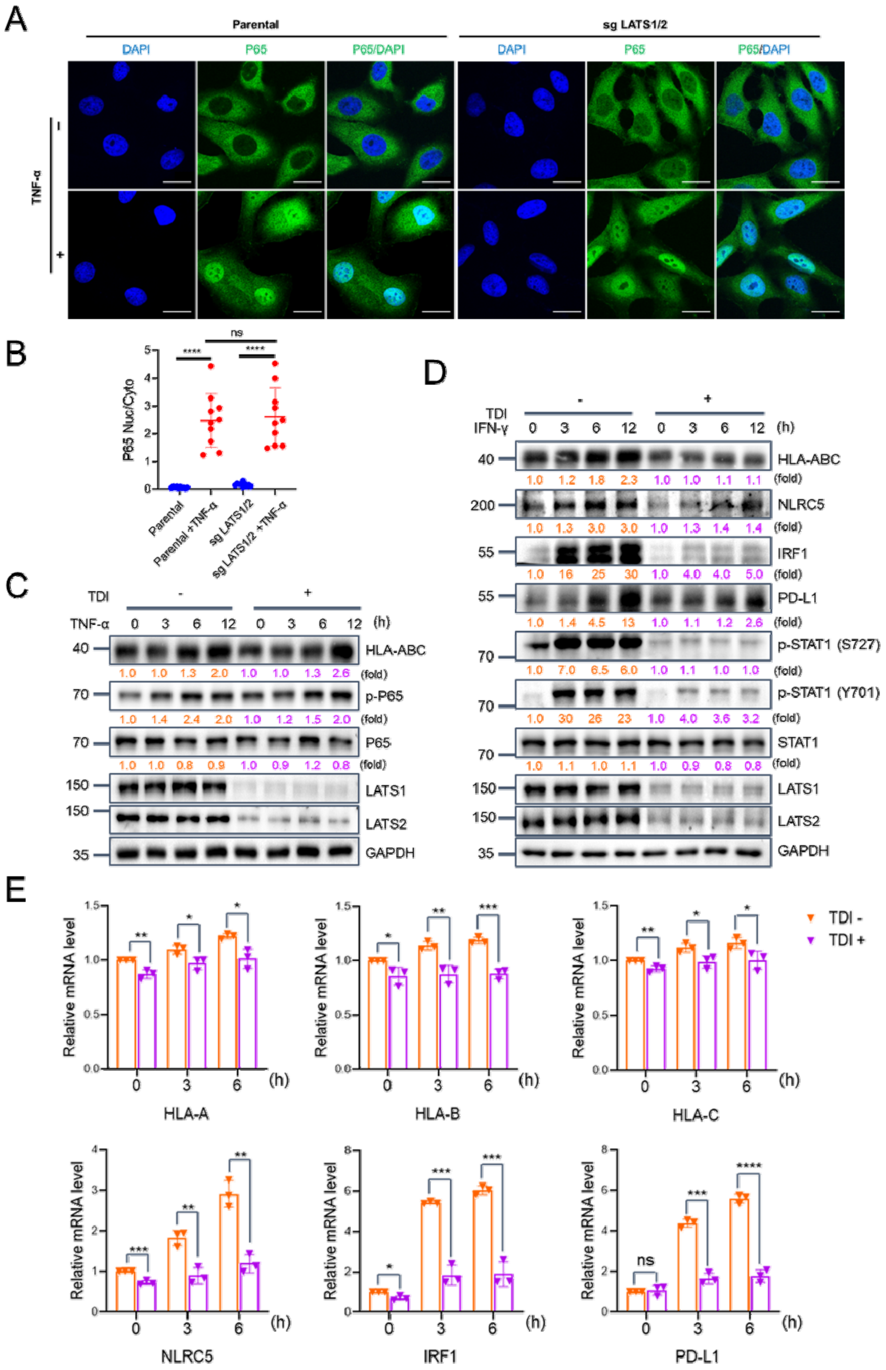

Fig. S4. Inhibition of LATS1/2 attenuates the transcriptional responses induced by IFN-γ (related to Fig. 3).

(A, B) Representative IF images from parental and LATS1/2-KO KLE cells treated with TNFα (20 ng/ml) for 6 h, stained with P65 and DAPI (A). Scale bar, 20 μm. Quantification of the ratio of P65 in Nuc/Cyto is shown in (B). Data are shown as means ± SD (n = 10).

(C) Western blotting of indicated proteins in the WCLs from SPEC-2 cells and LATS1/2

inhibited SPEC-2 cells by TDI-011536 (3 μM) for 24 h who were subsequently treated with TNFα (20 ng/ml) and harvested at different time points.

(D) Western blotting of indicated proteins in the WCLs from SPEC-2 cells and LATS1/2 inhibited SPEC-2 cells by TDI-011536 (3 μM) for 24 h who were subsequently treated with IFN-γ (30 ng/ml) and harvested at different time points.

(E) RT-qPCR measurement of the mRNA expression of IFN-γ target genes in SPEC-2 cells and LATS1/2 inhibited SPEC-2 cells by TDI-011536 (3 μM) for 24 h who were subsequently treated with IFN-γ (30 ng/ml) and harvested at different time points. Data are shown as means ± SD (n=3).

P values are calculated using One-way ANOVA test in (B) and the Multiple t-tests in (E). *p<0.05, **p<0.01, ***p<0.001, ****p<0.0001, ns: no significant.

Fig. S5

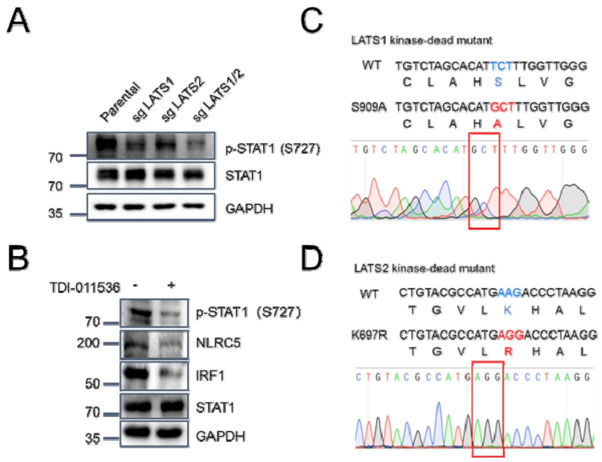

Fig. S5. LATS1/2 directly interacts with STAT1 and phosphorylates STAT1 (related to Fig. 4).

(A) Western blotting of the indicated proteins in the WCLs from parental, LATS1-KO, LATS2-KO or LATS1/2-KO HEC-1A cells.

(B) Western blotting of the indicated proteins in WCLs from HEC-1A parental cells treated with TDI-011536 (3 μM) for 24 h.

(C,D) Confirmation of the correct mutation of LATS1-S909A (C) and LATS2-K697R (D) by Sanger DNA sequencing.

Fig. S6

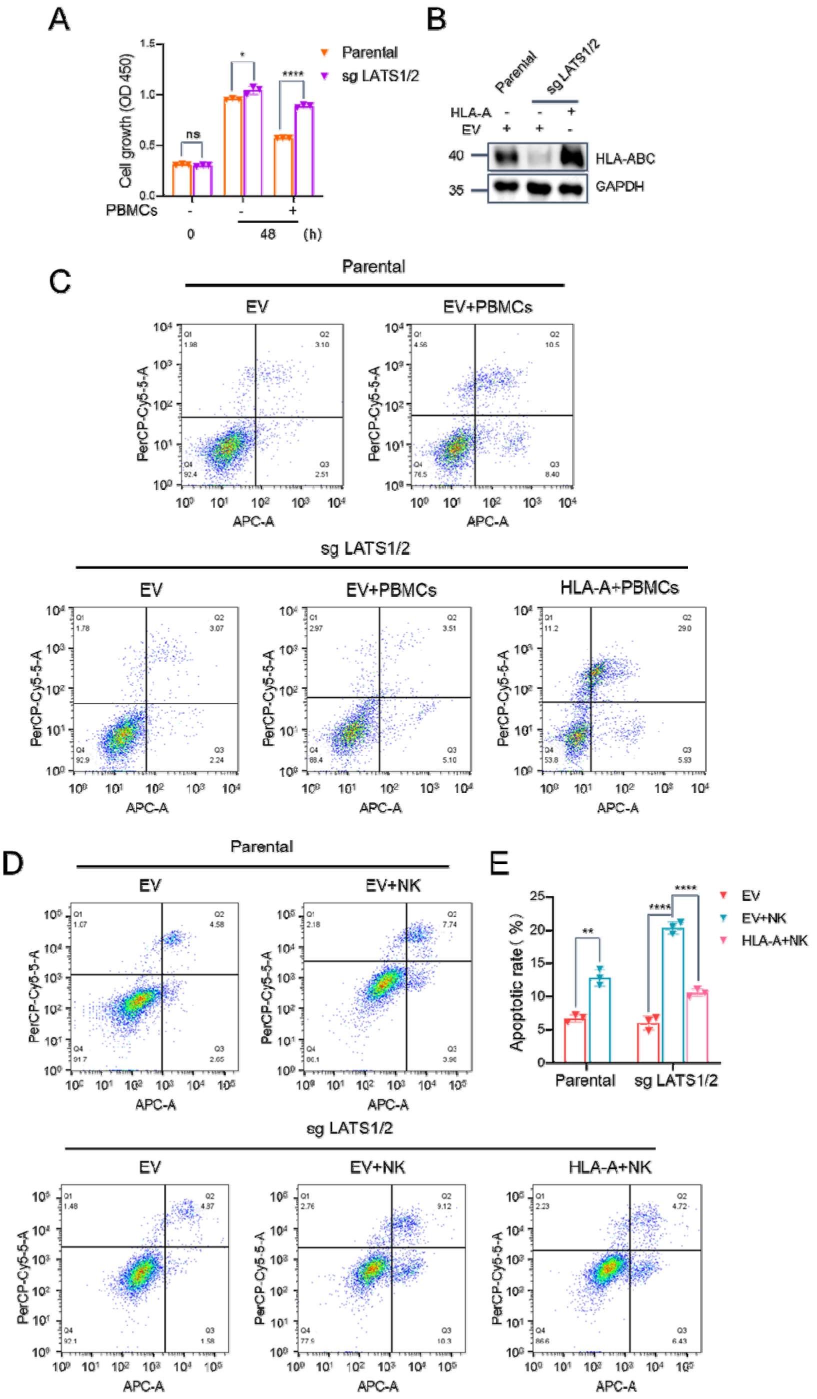

Fig. S6. LATS1/2 loss promotes tumor immune evasion by downregulating MHC-I expression (related to Fig. 5).

(A) CCK-8 cell proliferation analysis of parental and LATS1/2-KO HEC-1A cells co-cultured with PBMCs for 48 h.

(B) Exogenous HLA-A was reintroduced into LATS1/2-KO KLE cells to generate HLA-A reconstituted LATS1/2-KO KLE cells.

(C) Parental and LATS1/2-KO KLE cells or exogenous HLA-A reconstituted LATS1/2-KO KLE cells were co-cultured with PBMCs for 24 h. Flow cytometric analysis was performed to determine the apoptotic rates of KLE cells.

(D-E) Parental and LATS1/2-KO KLE cells or exogenous HLA-A reconstituted LATS1/2-KO KLE cells were co-cultured with NK cells for 24 h. Flow cytometric analysis was performed to determine the apoptotic rates of KLE cells. Data are shown as means ± SD (n=3).

P values are calculated using the Multiple t-tests in (A, E). *p<0.05, **p<0.01, ****p<0.0001, ns: no significant.

.
